# Supplementary material for: A UMLS-based spell checker for natural language processing in vaccine safety
Source: BMC Med Inform Decis Mak. 2007 Feb 12;7:3. doi: 10.1186/1472-6947-7-3 (PMC1805499; doi:10.1186/1472-6947-7-3)
Supplement: Additional file 13 — RAP application directory. Sets up directory for RAP (RDF application for PHP) source code [file 1472-6947-7-3-S13.gz › rap/doc/phpdoc/syntax/N3Parser.html]

xml version="1.0" encoding="iso-8859-1"?


Docs For Class N3Parser


## Class N3Parser

Description

Description |
Vars (details)
| Methods (details)

PHP Notation3 Parser

This parser can parse a subset of n3, reporting triples to a callback function or constructing a RAP Model ( http://www.wiwiss.fu-berlin.de/suhl/bizer/rdfapi )

Supported N3 features:

- Standard things, repeated triples ( ; and , ), blank nodes using [ ], self-reference ('<>')
- @prefix mappings
- = maps to owl#sameAs
- a maps to rdf-syntax-ns#type
- Literal datytype- and xmlLanguageTag support

Un-supported N3 Features include:

- Reification using { }
- . and ^ operators for tree traversal
- Any log operators, like log:forAll etc.

This parser is based on n3.py from Epp released 2nd March, 2002. by Sean B. Palmer ( http://infomesh.net/2002/eep/20020302-013802/n3.py )

This parser is released under the GNU GPL license. ( http://www.gnu.org/licenses/gpl.txt )

**History:**

- 04-05-2005 toke() function improved by Hannes Gassert hannes.gassert@deri.org
- 03-25-2005 N3 list processing added by Hannes Gassert hannes.gassert@deri.org
- 12-06-2004 improved namespace handling added (tobias.gauss@web.de)
- 08-10-2004 Function for converting strings to its unicode NFC form. Benjamin Nowack <bnowack@appmosphere.com>
- 10-05-2004 Fixed bug with trailing space on qnames and space before ] parsin bug
- 11-27-2003 fixed problems with whithespaces at the end of bNodes
- 11-18-2003 Changed xml:language regex for supporting lang-tags like en-uk.
- 11-07-2003 Added "setFixBnodes" function. Sets, if Bnodes should be renamed to the BNODE\_PREFIX constant.
- 10-27-2003 fixed problems in generateModel(), changed regEx for Literals.
- 10-24-2003 Added support for Literals with rdf:DataType and xml:Language Tags. URI-Self-Reference with '<>' is supported.
- 08-01-2003 Made compatible with new v6 MemModel.
- 07-31-2003 Function generateModel() added.
- 07-16-2003 Fixed bug with anon nodes alone on a line.
- 06-08-2003 Initial version converted from n3.py.

- access: public
- version: V0.9.1
- author: Sean B. Palmer <sean@mysterylights.com>, Gunnar AA. Grimnes <ggrimnes@csd.abdn.ac.uk>, Daniel Westphal <mail@d-westphal.de>

Located in /syntax/N3Parser.php (line 61)

```
Object
   |
   --N3Parser
```

Variable Summary

Description |
Vars (details)
|
Methods (details)

mixed
$bNode

mixed
$DAML\_NS

mixed
$debug

mixed
$OWL\_NS

mixed
$parsedNamespaces

mixed
$parseError

mixed
$RDF\_NS

mixed
$Tokens

Method Summary

Description |
Vars (details)
|
Methods (details)

N3Parser
N3Parser
()

object MemModel
&generateModel
($path $path, [mixed $dummy = false], [mixed $model = false])

array
getPovs
(array $list)

void
parse
(string $s)

object Model
parse2model
(string $s, [mixed $model = false])

void
setFixBnodes
(boolean $set)

void
uparse
(string $s, string $func)

Variables

Description |
Vars (details)
|
Methods (details)

mixed
$bNode
(line 67)

mixed
$DAML\_NS
(line 68)

mixed
$debug
(line 69)

mixed
$OWL\_NS
(line 68)

mixed
$parsedNamespaces
= array() (line 71)

mixed
$parseError
(line 70)

mixed
$RDF\_NS
(line 68)

mixed
$Tokens
(line 66)

Methods

Description |
Vars (details)
Methods (details)

Constructor N3Parser (line 79)

Constructor

- access: public

N3Parser

N3Parser
()

generateModel (line 223)

Generate a new MemModel from an URI or file.

- throws: PhpError
- access: public

object MemModel

&generateModel
($path $path, [mixed $dummy = false], [mixed $model = false])

- $path
  $path

getPovs (line 659)

Gets a list of triples with same subject e.g. :Gunnar :firstname "Gunnar" ; :lastname "Grimnes.

array

getPovs
(array $list)

- array
  $list

parse (line 132)

This parses a N3 string and prints out the triples

- access: public

void

parse
(string $s)

- string
  $s

parse2model (line 189)

This parses a N3 string and returns a memmodel

- access: public

object Model

parse2model
(string $s, [mixed $model = false])

- string
  $s

setFixBnodes (line 121)

Sets, if BlankNode labels should be replaced by the generic label from the constants.php file

default is "false" -> the used label in n3 is parsed to the model

- access: public

void

setFixBnodes
(boolean $set)

- boolean
  $set

uparse (line 160)

This parses a N3 string and calls func($subject, $predicate, $object) with each trioke

- access: public

void

uparse
(string $s, string $func)

- string
  $s
- string
  $func

#### Inherited Methods


Inherited From Object

> Object::toString()

Documentation generated on Thu, 7 Jul 2005 13:42:12 +0200 by phpDocumentor 1.3.0RC3
